# Anti-cancer activity of Ginger (*Zingiber officinale*) leaf through the expression of activating transcription factor 3 in human colorectal cancer cells

**DOI:** 10.1186/1472-6882-14-408

**Published:** 2014-10-23

**Authors:** Gwang Hun Park, Jae Ho Park, Hun Min Song, Hyun Ji Eo, Mi Kyoung Kim, Jin Wook Lee, Man Hyo Lee, Kiu-Hyung Cho, Jeong Rak Lee, Hyeon Je Cho, Jin Boo Jeong

**Affiliations:** Department of Bioresource Sciences, Andong National University, Andong, 760749 South Korea; Department of Medicinal Plant Science, Jungwon University, Goesan, 367805 South Korea; Gyeongbuk Institute for Bio-industry, Andong, 760380 South Korea; Insititute of Agricultural Science and Technology, Andong National University, Andong, 760749 South Korea; Department of Medicinal Plant Resources, Andong National University, Andong, 760749 South Korea

**Keywords:** Ginger leaf, Cancer chemoprevention, Activating transcription factor 3, Apoptosis, Colorectal cancer

## Abstract

**Background:**

Ginger leaf (GL) has long been used as a vegetable, tea and herbal medicine. However, its pharmacological properties are still poorly understood. Thus, we performed *in vitro* studies to evaluate anti-cancer properties of ginger leaf and then elucidate the potential mechanisms involved.

**Methods:**

Cell viability was measured by MTT assay. ATF3 expression level was evaluated by Western blot or RT-PCR and ATF3 transcriptional activity was determined using a dual-luciferase assay kit after the transfection of ATF3 promoter constructs. In addition, ATF3-dependent apoptosis was evaluated by Western blot after ATF3 knockdown using ATF3 siRNA.

**Results:**

Exposure of GL to human colorectal cancer cells (HCT116, SW480 and LoVo cells) reduced the cell viability and induced apoptosis in a dose-dependent manner. In addition, GL reduced cell viability in MCF-7, MDA-MB-231 and HepG-2 cells. ATF3 knockdown attenuated GL-mediated apoptosis. GL increased activating transcription factor 3 (ATF3) expressions in both protein and mRNA level and activated ATF3 promoter activity, indicating transcriptional activation of ATF3 gene by GL. In addition, our data showed that GL-responsible sites might be between -318 and -85 region of the ATF3 promoter. We also observed that ERK1/2 inhibition by PD98059 attenuated GL-mediated ATF3 expression but not p38 inhibition by SB203580, indicating ERK1/2 pathway implicated in GL-induced ATF3 activation.

**Conclusions:**

These findings suggest that the reduction of cell viability and apoptosis by GL may be a result of ATF3 promoter activation and subsequent increase of ATF3 expression through ERK1/2 activation in human colorectal cancer cells.

## Background

Cancer is a major problem of public health in many other parts of the world including the United States [1]. Among the kinds of cancers, human colorectal cancer is the third leading cause of cancer-related death in both male and females in the United States [1]. Although surgery and chemotherapy have been the most common treatment for colorectal cancer, cancer chemoprevention with dietary factors has received attention as the most effective approach to reduce colorectal cancer-related mortality. Thus, for the last two decades, many researchers have tested and reported anti-cancer activities of natural products in dietary factors such as fruits, vegetables and teas [2, 3].

Gingers as perennial herbs belonging to the family Zingiberaceae have been widely used as spices, condiments and herbal medicine for treatment of cold, fever, headache, nausea and digestive problems [4]. Ginger and its general compounds such as gingerols, shogaols, paradols and zingerone exert immuno-modulatory, anti-apoptotic, anti-tumourigenic, anti-inflammatory, anti-hyperglycaemic, anti-hyperlipidaemic, antioxidant and anti-emetic activities [4]. Ginger leaves have also been used for food flavouring and traditional medicine [5]. Past pharmacological studies of ginger were confined to rhizomes. Thus, in light of the pharmacological actions of ginger leaves, this study was performed to investigate the anti-cancer activity and elucidate the potential mechanism by which ginger leaves induces the reduction of cell viability and apoptosis in human colorectal cancer cells. Here, for the first time, we report that ginger leaves leads to transcriptional activation of activating transcription factor 3 (ATF3) which may be associated with the reduction of cell viability and induction of apoptosis in human colorectal cancer cells.

## Methods

### Materials

Cell culture media, Dulbecco’s Modified Eagle medium (DMEM)/F-12 1:1 Modified medium (DMEM/F-12) was purchased from Lonza (Walkersville, MD, USA). The 3-(4,5-dimethylthizaol-2-yl)-2,5-diphenyl tetrazolium bromide (MTT) and SP600125 was purchased from Sigma Aldrich (St. Louis, MO, USA). SB203580 and PD98059 were purchased from Calbiochem (San Diego, CA, USA). ATF3 antibody and ATF3 siRNA were provided from Santa Cruz Biotechnology, Inc (Santa Cruz, CA, USA). Antibodies against β-actin and poly (ADP-ribose) polymerase (PARP), and control siRNA were purchased from Cell Signaling (Bervely, MA, USA). ATF3 promoter constructs (-1420/+34, -718/+34, -514/+34, -318/+34, -147/+34 and -84/+34) were kindly provided by Dr. S-H Lee (University of Maryland College Park, MD, USA). All chemicals were purchased from Fisher Scientific, unless otherwise specified.

### Sample preparation

The leaves of ginger (*Zingiber officinale,* voucher number: PARK1003(ANH)) were kindly provided by the Bonghwa Alpine Medicinal Plant Experiment Station, Korea. One kilogram of ginger leaf was extracted with 1000 ml of 80% methanol with shaking for 24 h. After 24 h, the methanol-soluble fraction was filtered and concentrated to approximately 20 ml volume using a vacuum evaporator and then fractionated with petroleum ether and ethyl acetate in a separating funnel. The ethyl acetate fraction was separated from the mixture, evaporated by a vacuum evaporator, and prepared aseptically and kept in a refrigerator.

### Cell culture and treatment

Human colorectal cancer cell lines (HCT116, SW480 and LoVo), human breast cancer cell lines (MCF-7 and MDA-MB231) and human hepatocellular carcinoma (HepG-2) were purchased from Korean Cell Line Bank (Seoul, Korea) and grown in DMEM/F-12 supplemented with 10% fatal bovine serum (FBS), 100 U/ml penicillin and 100 μg/ml streptomycin. The cells were maintained at 37°C under a humidified atmosphere of 5% CO_2_. The extracts of ginger leaf (GL) were dissolved in dimethyl sulfoxide (DMSO) and treated to cells. DMSO was used as a vehicle and the final DMSO concentration did not exceed 0.1% (v/v).

### Cell viability

Cell viability was measured using MTT assay system. Briefly, cells were plated onto 96-well plated and grown overnight. The cells were treated with 0, 50, 100 and 200 μg/ml of GL for 24 and 48 h. Then, the cells were incubated with 50 μl of MTT solution (1 mg/ml) for an additional 2 h. The resulting crystals were dissolved in DMSO. The formation of formazan was measured by reading absorbance at a wavelength of 570 nm.

### Reverse transcriptase-polymerase chain reaction (RT-PCR)

Total RNA was prepared using a RNeasy Mini Kit (Qiagen, Valencia, CA, USA) and total RNA (1 μg) was reverse-transcribed using a Verso cDNA Kit (Thermo Scientific, Pittsburgh, PA, USA) according to the manufacturer’s protocol for cDNA synthesis. PCR was carried out using PCR Master Mix Kit (Promega, Madison, WI, USA) with primers for human ATF3 and human GAPDH as follows: human ATF3: 5′-gtttgaggattttgctaacctgac-3′, and reverse 5′-agctgcaatcttatttctttctcgt-3′; huaman GAPDH: forward 5′-acccagaagactgtggatgg-3′ and reverse 5′-ttctagacggcaggtcaggt-3′.

### Transient transfections

Transient transfections were performed using the PolyJet DNA transfection reagent (SignaGen Laboratories, Ijamsville, MD, USA) according to the manufacturers’ instruction. HCT116 and SW480 cells were plated in 12-well plates at a concentration of 2 × 105 cells/well. After growth overnight, plasmid mixtures containing 0.5 μg of ATF3 promoter linked to luciferase and 0.05 μg of *pRL-null* vector were transfected for 24 h. The transfected cells were cultured in the absence or presence of GL for the indicated times. The cells were then harvested in 1 × luciferase lysis buffer, and luciferase activity was normalized to the *pRL-null* luciferase activity using a dual-luciferase assay kit (Promega, Madison, WI, USA).

### Transfection of small interference RNA (siRNA)

The cells were plated in six-well plates and incubated overnight. HCT116 cells were transfected with control siRNA and ATF3 siRNA for 48 h at a concentration of 100 nM using TransIT-TKO transfection reagent (Mirus, Madison, WI, USA) according to the manufacturer’s instruction. Then the cells were treated with GL (100 μg/ml) for 24 h.

### Cell death assay

Cell death was performed using Cell Death Detection ELISA^PLUS^ Kit (Roche Diagnostics, Indianapolis, IN, USA) according to the manufacturer’s instruction. Briefly, HCT116 and SW480 cells were seeded in 12-well plate. After 24 h, cells were treated with 0, 25, 50 and 100 μM of GL for 24 h. Cytosol was prepared using Nuclear Extract Kit (Active Motif, Carlsbad, CA, USA). Equal amounts of cytosolic extracts, immunoreagent containing anti-histone-biotin, and anti-DNA-POD were added to microplate well and incubated for 2 h under shaking. After washing, the ABTS solution was added to each well for 20 min and then the ABTS stop solution was added. The absorbance was recorded at 405 nm and 490.

### SDS-PAGE and Western blot

After GL treatment, cells were washed with 1 × phosphate-buffered saline (PBS), and lysed in radioimmunoprecipitation assay (RIPA) buffer (Boston Bio Products, Ashland, MA, USA) supplemented with protease inhibitor cocktail (Sigma-Aldrich, St. Louis, MD. USA) and phosphatase inhibitor cocktail (Sigma-Aldrich), and centrifuged at 15,000 × g for 10 min at 4°C. Protein concentration was determined by the bicinchoninic acid (BCA) protein assay (Pierce, Rockford, IL, USA). The proteins were separated on SDS-PAGE and transferred to PVDF membrane (Bio-Rad Laboratories, Inc., Hercules, CA, USA). The membranes were blocked for non-specific binding with 5% non-fat dry milk in Tris-buffered saline containing 0.05% Tween 20 (TBS-T) for 1 h at room temperature and then incubated with specific primary antibodies in 5% non-fat dry milk at 4°C overnight. After three washes with TBS-T, the blots were incubated with horse radish peroxidase (HRP)-conjugated immunoglobulin G (IgG) for 1 h at room temperature and chemiluminescence was detected with ECL Western blotting substrate (Amersham Biosciences, Piscataway, NJ, USA) and visualized in Polaroid film.

### Statistical analysis

Statistical analysis was performed with the Student’s unpaired *t*-test, with statistical significance set at *, P <0.05.

## Results

### Effect of GL on cell viability and apoptosis

**Figure 1:**
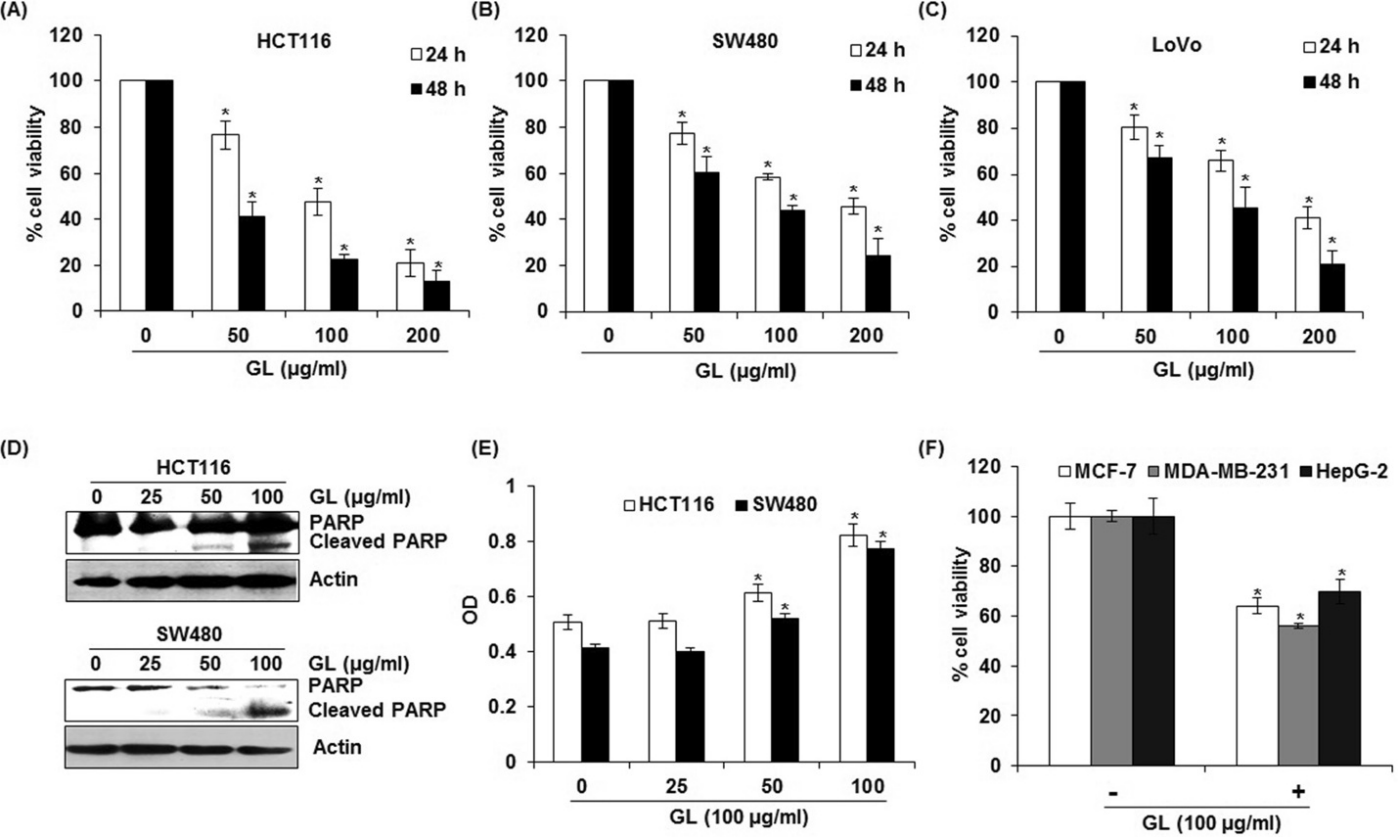
**The effects of GL on cell viability and apoptosis in human colorectal cancer cells.** HCT116 **(A)**, SW480 **(B)** and LoVo cells **(C)** were treated with 0, 50, 100 and 200 μg/ml of GL for 24 and 48 h. Cell viability was measured using MTT assay system and expressed as % cell viability compared to the cell without GL treatment. *P < 0.05 compared to cells without GL treatment. **(D)** HCT116 and SW480 cells were treated with 0, 25, 50 and 100 μg/ml of GL for 24 h. Cell lysates were subjected to SDS-PAGE and the Western blot was performed using antibodies against PARP. Actin was used as internal control. **(E)** HCT116 and SW480 cells were treated with 0, 25, 50 or 100 μM of GL for 24 h. the cytosol fraction was extracted from GL-treated cells, and the cell death was measured using the Cell Death Detection ELISA^PLUS^ Kit, and expressed as absorbance (A_405_-A_490_). *p < 0.05 compared to cells without GL treatment. **(F)** MCF-7, MDA-MB-231 and HepG-2 cells were treated with 100 μg/ml of GL for 24 h. Cell viability was measured using MTT assay system and expressed as % cell viability compared to the cell without GL treatment. *P < 0.05 compared to cells without GL treatment.

To investigate whether ginger leaf affects the cell viability in human colorectal cancer cells, HCT116, SW480 and LoVo, the cells were incubated with 50, 100 and 200 μg/ml of GL for 24 and 48 h, and cell viability was measured using MTT assay. As shown in Figure 1A, GL reduced the viability of HCT116 cells by 24 and 59% at 50 μg/ml, 53 and 79% at 100 μg/ml, and 79 and 88% at 200 μg/ml at 24 and 48 h after GL treatment, respectively. We also found that the viability of SW480 cells was reduced by GL treatment for 24 and 48 h by 23 and 40% at 50 μg/ml, 42 and 57% at 100 μg/ml, and 55 and 76% at 200 μg/ml, respectively (Figure 1B). In addition, GL suppressed LoVo cell viability by 20 and 33% at 50 μg/ml, 34 and 55% at 100 μg/ml, and 59 and 80% at 200 μg/ml after GL treatment for 24 and 48 h, respectively (Figure 1C). HCT116 and SW480 cells were treated with 25, 50 and 100 μg/ml of GL for 24 h, and apoptosis was measured using Western blot against cleaved PARP and the cell death assay using ELISA-based cell death kit. As shown in Figure 1D, the cleavage of PARP was observed at 50 and 100 μg/ml in HCT116 and SW480 cells, respectively, which indicates that GL may induce apoptosis. In addition, significant increases of cell death were observed in the cells treated with GL (Figure 1E). To determine if GL shows a reduction of cell viability in other types of cancer cells, breast (MCF-7 and MDA-MD-231) and hepatocellular carcinoma (HepG-2) cells were treated with 100 μg/ml at 24 h. As a result, GL-treatment for 24 h reduced the cell viability by 36% in MCF-7, 44% in MDA-MB-231, and 30% in HepG-2 cells, respectively (Figure 1F). These data indicates that GL may have anti-cancer activity in colorectal cancer, breast cancer and hepatocellular carcinoma cells.

### Effect of GL on ATF3 expression in HCT116 and SW480 cells

**Figure 2:**
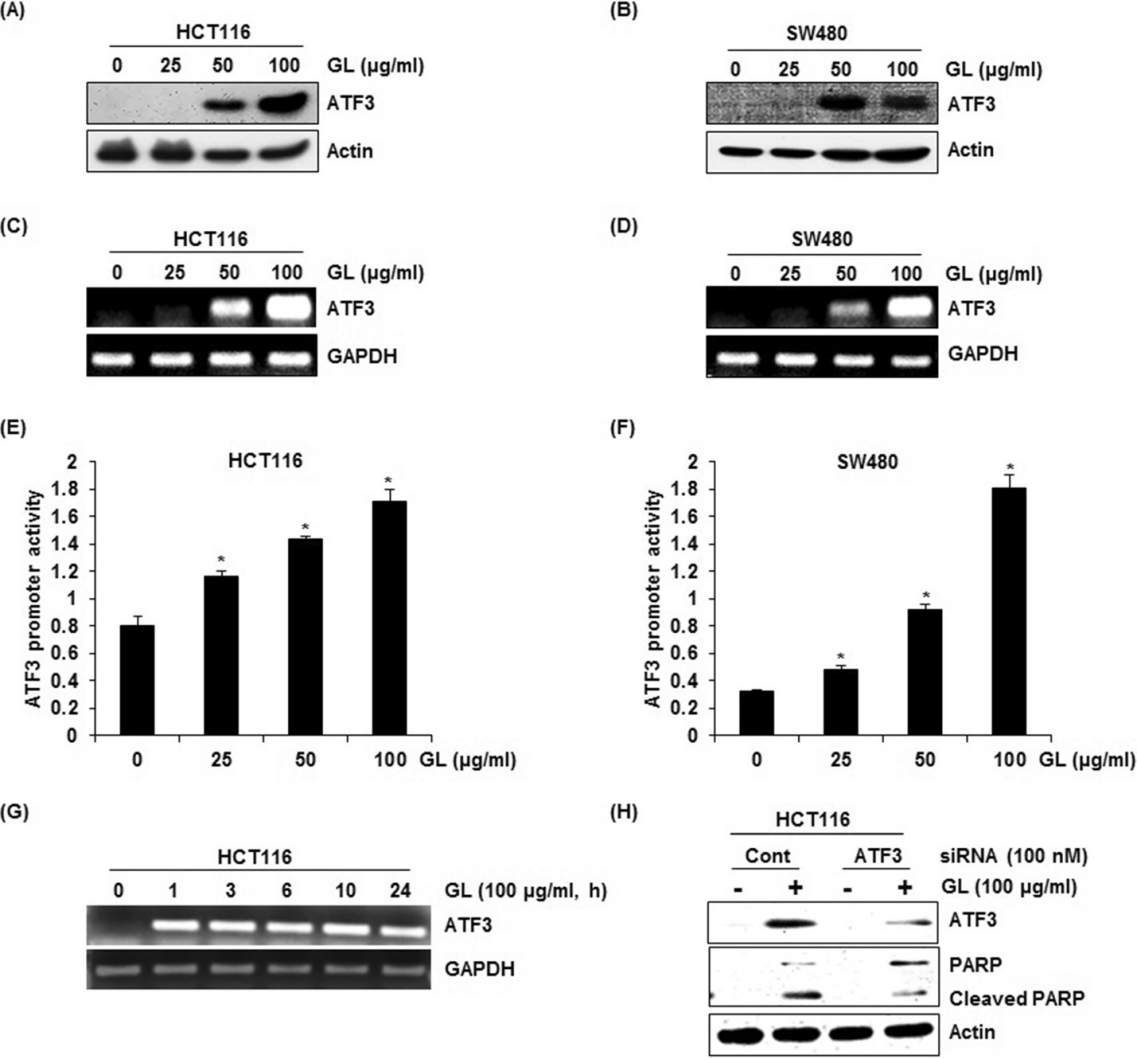
**Effects of GL on ATF3 activation in human colorectal cancer cells. (A, B)** HCT116 and SW480 cells were treated with 0, 25, 50 and 100 μg/ml of GL for 24 h. Cell lysates were subjected to SDS-PAGE and the Western blot was performed using antibodies against ATF3. Actin was used as internal control. For RT-PCR analysis of ATF3 gene expression, total RNA was prepared after GL treatment for 24 h in dose-course experiments **(C, D)** or for the indicated times in time-course experiments **(G)**. GAPDH were used as internal control. For ATF3 promoter activity, luciferase construct containing -1420 to +34 of human ATF3 promoter region was cotransfected with pRL-null vector and the cells were treated with GL for 24 h in dose-course experiments **(E, F)** and luciferase activity was measured. *P <0.05 compared to cells without GL treatment. **(H)** ATF3 siRNA was transfected into HCT116 for 48 h and then GL was treated for 24 h. Cell lysates were subjected to SDS-PAGE the Western blot was performed using antibodies against PARP. Actin was used as internal control.

Since there is growing evidence that ATF3 is associated with to cell growth arrest and apoptosis in human colorectal cancer cells [6], we evaluated whether GL affects ATF3 expression in HCT116 and SW480 cells. As shown in Figure 2A-D, GL-induced ATF3 expressions were observed at 50 and 100 μg/ml of GL in both protein and mRNA level. To investigate whether increase in ATF3 expression by GL treatment was mediated from transcription activation of ATF3 promoter, ATF3 promoter (-1420/+34) transfected HCT116 and SW480 cells were incubated with 25, 50 and 100 μg/ml of GL for 24 h. As shown in Figure 2E and F, GL dose-dependently activated ATF3 transcription. In time-course experiments, we found that the expression of ATF3 mRNA activation by GL started to be increased at 1 h after GL treatment in HCT116 cells (Figure 2G). To see whether ATF3 expression affect GL-mediated apoptosis, cleaved PARP was measured by Western blot in ATF3 knock downed-HCT116 cells. As shown in Figure 2H, knockdown of ATF3 by ATF3 siRNA reduced the cleavage of PARP by GL, indicating that ATF3 may be one of important genes in GL-mediated apoptosis.

### Kinases affecting GL-induced ATF3 activation

**Figure 3:**
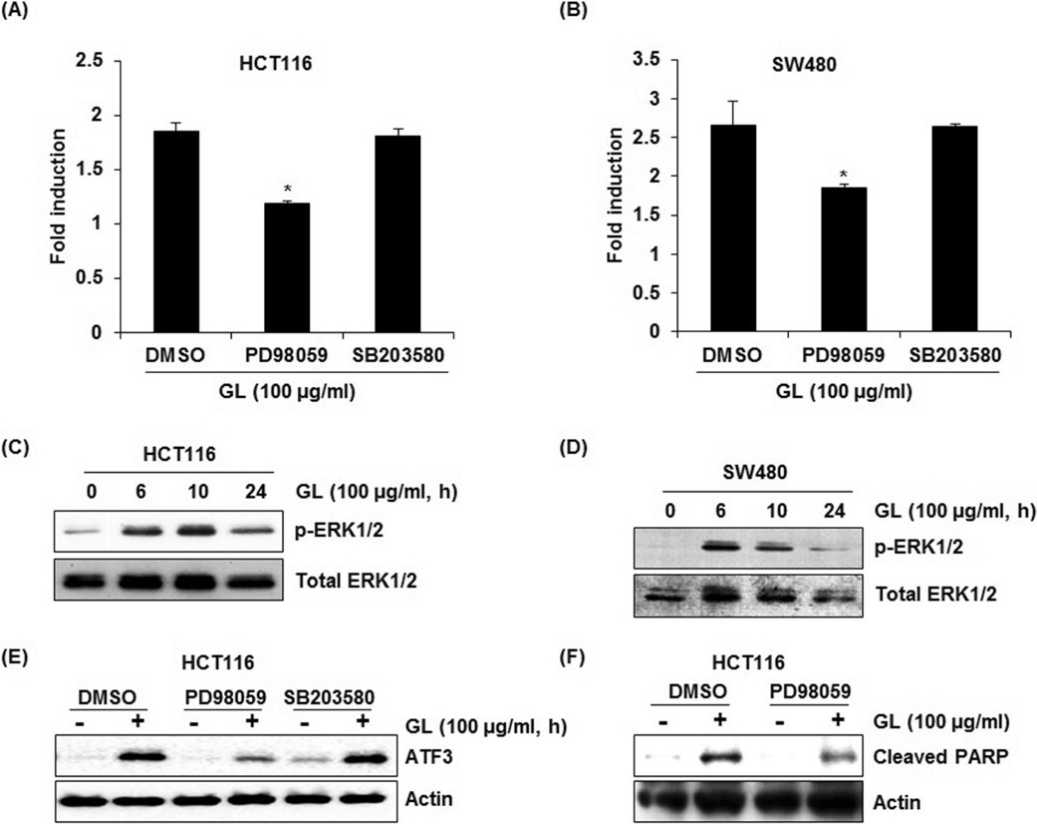
**Up-stream signaling pathways affecting GL-mediated ATF3 activation. (A, B)** Luciferase construct containing -1420 to +34 of human ATF3 promoter region was cotransfected with pRL-null vector. Then, the cells were pretreated with 20 μM of PD98059 (ERK1/2 inhibitor) or SB203580 (p38 inhibitor) and then cotreated with 100 μg/ml of GL for 24 h. *P <0.05 compared to cells without the treatment of inhibitors. **(C, D)** HCT116 and SW480 cells were treated with 100 μg/ml of GL for indicated times. Cell lysates were subjected to SDS-PAGE and the Western blot was performed using antibodies against p-ERK1/2 and ERK1/2. Actin was used as internal control. **(E)** HCT116 cells were pre-treated with 20 μM of PD98059 (ERK1/2 inhibitor) or SB203580 (p38 inhibitor) and then co-treated with 100 μg/ml of EAFAD-B for 6 h. Cell lysates were subjected to SDS-PAGE the Western blot was performed using antibodies against ATF3. Actin was used as internal control. **(F)** HCT116 cells were pretreated with 20 μM of PD98059 (ERK1/2 inhibitor) for 2 h and then co-treated with 100 μM of GL for 24 h. Cell lysates were subjected to SDS-PAGE the Western blot was performed using antibodies against PARP. Actin was used as internal control.

To determine upstream kinase affecting GL-induced ATF3 transcriptional activation, ATF3 promoter (-1420/+34) transfected HCT116 and SW480 cells were pretreated with 20 μM of PD98059 (ERK1/2 inhibitor) and SB203580 (p38 inhibitor) for 2 h and co-treated with 100 μg/ml of GL for 24 h. As shown in Figure 3A and B, ERK1/2 inhibition by PD98059 attenuated GL-mediated ATF3 promoter activation, while p38 inhibition by SB203580 did not affect ATF3 activation by GL in both HCT116 and SW480 cells. We also investigated whether JNK signaling affects GL-mediated ATF3 promoter activation and found that JNK inhibition by SP600125 did not affect ATF3 promoter activation by GL (data not shown).

We investigated that GL treatment affects ERK1/2 activation. As shown in Figure 3C and D, GL enhanced phosphorylation of ERK1/2 protein. We also tested that the inhibitions of ERK1/2 by PD98059 and p38MAPK by SB203580 affect GL-mediated ATF3 protein level. In these experiments, the inhibition of ERK1/2 by PD98059 ameliorated the increase of ATF3 protein level by GL, while p38 MAPK inhibition did not affect ATF3 protein level in HCT116 cells (Figure 3E). Because GL-mediated ATF3 activation was affected by ERK1/2, we evaluated if ERK1/2 affects GL-mediated apoptosis. As a result, ERK1/2 inhibition by PD98059 attenuated GL-mediated cleavage of PARP in HCT116 cells (Figure 3F). These results indicates that GL-mediated apoptosis may result from ERK1/2-dependent ATF3 activation.

### Identification of ATF3 promoter sites responsible for GL-induced ATF3 activation

**Figure 4:**
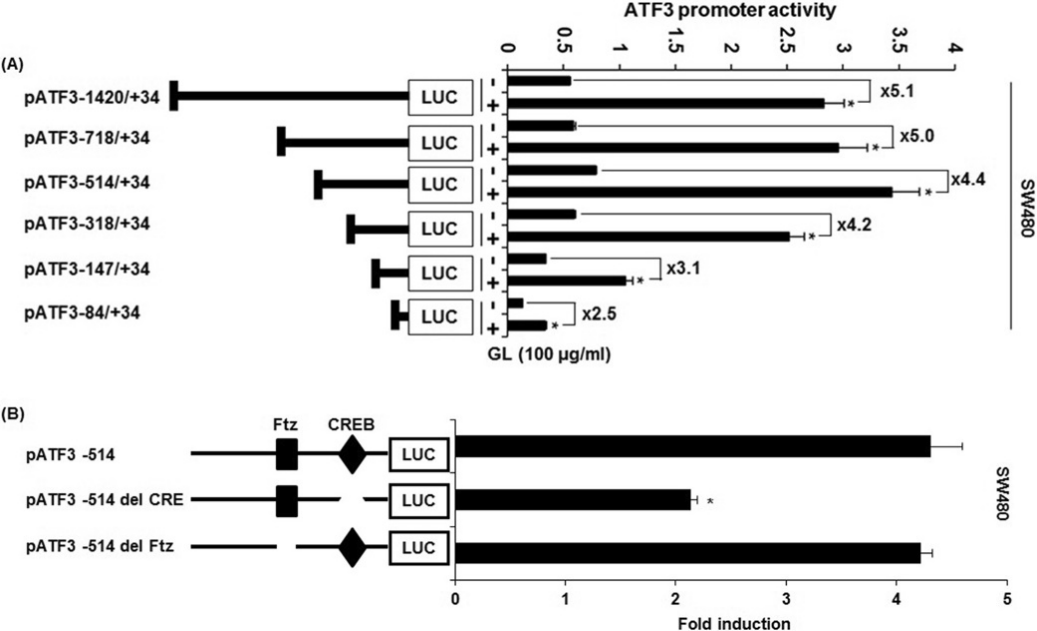
**Identification of ATF3 promoter sites responsible for GL-induced ATF3 activation. (A)**. Each indicated construct of the ATF3 promoter (0.5 μg) was co-transfected with 0.05 μg of pRL-null vector into SW480 cells, and cells were treated with 100 μg/ml of GL for 24 h. **(B)** Each deletion construct (0.5 μg) of the ATF3 promoter was co-transfected with 0.05 μg of pRL-null vector into SW480 cells and cells were treated with 100 μg/ml of GL for 24 h. Luciferase activity was measured. *P <0.05 compared to cells without GL treatment.

To investigate ATF3 promoter sites which can be responsible for GL-induced ATF3 activation, promoter activity was measured using different sizes of ATF3 promoter luciferase constructs (-1420/+34, -718/+34, -514/+34, -318/+34, -147/+34 and -84/+34). These constructs were transfected into SW480 cells and treated with 100 μg/ml of GL for 24 h. As shown in Figure 4A, GL treatment resulted in an increase of promoter activity. The fold induction in SW480 cells was 5.1, 5.0, 4.4, 4.2, 3.1 and 2.5 in pATF3-1420/+34, pATF3- 718/+ 34, pATF3- 514/+ 34, pATF3- 318/+ 34, pATF3-147/+ 34, and pATF3- 84/+ 34, respectively. Because GL increased ATF3 promoter activity more than 4.0-fold in SW480 cells, GL-responsible sites might be between -318 and -85 region of the ATF3 promoter. The Fushi tarazu (Ftz) and CREB is *cis*-acting elements in ATF3 promoter containing -147 and -85 by program (Gene Regulation, TFSEARCH, and Transcription Element System). To identify the role of each *cis*-acting element, each site-deleted ATF3 promoter constructs were transfected into SW480 cells and treated with 100 μg/ml of GL for 24 h. As shown in Figure 4B, GL-induced ATF3 promoter activity was significantly decreased when the CREB site was deleted. However, the deletion of Ftz sites did not affect ATF3 promoter activity by GL. These data indicated that CREB is an important region in GL-induced ATF3 expression.

## Discussion

Because many dietary factors exert anti-cancer activities, cancer chomoprevention with dietary factors has received attention as the most effective approach to reduce colorectal cancer-related mortality. Ginger leaves have been used as the dietary factors such a vegetable and tea, and the herbal medicine [5]. However, pharmacological actions of ginger leaves have not been studied. Here, we evaluated the anti-cancer activity of ginger leaves and elucidated its potential mechanism. In this study, we, for the first time, report that ginger leaves showed an anti-cancer activity associated with ATF3 activation in colorectal cancer cells,

ATF3, an ATF/CREB subfamily member, contains the basic-leucine zipper (b-ZIP) DNA binding domain [7]. ATF3 is dramatically expressed in response to several stresses in many different tissues and exerts diverse biological effects [7]. In cancer development, ATF3 exerts pro-or anti-apoptotic activities dependent on cell or tissue context [8, 9]. ATF3 expression was suppressed in human colorectal cancer [10] and expression of ATF3 induced apoptosis, growth arrest of colorectal cancer cells and *Ras*-stimulated tumourigenesis [11–13]. On the other hand, ATF3 induces DNA synthesis and expression of cyclin D1 in hepatocellular carcinoma cells [14] and enhances cancer cell-initiating features in breast cancer [15]. Although ATF3 has dual effects in cancer development, ATF3 has been regarded as a major target of cancer chemoprevention in colorectal cancer. Our data indicate that GL increased ATF3 expression in both protein and mRNA level in a time- and dose-dependent manner through the activation of ATF3 promoter. In addition, it was reported that anti-cancer agents such as indole-3-carbinol [16], conjugated linoleic acid [17], epicatechin gallate [18], tolfenamic acid [19] and PI3 kinase inhibitor [13] induce ATF3-dependent apoptosis in colorectal cancer cells. In our study, GL increased the PARP cleavage and reduced the viability of colorectal cancer cells, indicating that increased apoptosis and reduction of cell viability may be mediated by activation of ATF3 expression in GL-treated cells.

There is a growing body of evidence to suggest that MAPK signaling is an important pathway regulating ATF3 expression [18, 20]. Therefore, we examined whether GL-mediated ATF3 activation is associated with the activation of ERK1/2, p38 and JNK. ERK1/2 inhibition by PD98059 attenuated GL-induced activation of ATF3 promoter and ATF3 expression but not in inhibition of p38 and JNK by SB203580 and bySP600125, indicating that ERK1/2 activation may contribute to GL-induced ATF3 activation. In addition, inhibition of ERK1/2 ameliorated GL-mediated apoptosis. In Western blot analysis for phosphorylation of ERK1/2, we found that GL induced a prolonged activation of ERK1/2. Why would late phase ERK activation correlate with proliferation and apoptosis remains to be understood. However, there is one hypothesis that prolonged activation of ERK1/2 can promote accumulation of p21^cip1^ resulting in cell cycle arrest and apoptosis [21]. Similarly, several anti-cancer agents have been reported to induce a prolonged activation of ERK1/2, which results in promoting apoptosis [22–25].

Interestingly, we found that GL-responsible sites for ATF3 activation might be between -318 and -85 region of the ATF3 promoter. ATF3 promoter includes various response elements such as AP-1, ATF/CRE, NF-κB, E2F and Myc/Max binding sites [26] and especially, EGR-1, CRE and Ftz are cis-acting elements in ATF3 promoter (-318/-85) [27] from which our data showed that GL-induced ATF3 promoter activity was significantly decreased when the CREB site was deleted. These data indicated that CREB is an important region in GL-induced ATF3 expression.

There is a report that ginger leaves has various bioactive compounds including quercetin, rutin, epicatechin, catechin, kaempferol, naringenin, salicylic acid, cinnamic acid, flavonoids and phenolics [28]. Among the bioactive compounds, quercetin has been reported to induce ATF3 expression in human colorectal cancer cells, Caco-2 [29]. Interestingly, we found that ginger leaves had more quercetin (2.124 mg/g dry weight) than ginger rhizoma (1.105 mg/g dry weight), which is similar to the previous report [28]. According to the previous study [30, 31] quercetin can be efficiently hydrolysed and absorbed in the intestinal lumen and plasma. Therefore, it is thought that quercetin may be responsible for ATF3-mediated apoptosis by ginger leaves in human colorectal cancer cells and quercetin of GL may be absorbed in the intestinal lumen and plasma of animal.

## Conclusions

In conclusion, ginger leaves may induce apoptosis and reduction of cell viability, followed by the increased ATF3 expression via activating ATF3 promoter in human colorectal cancer cells. In addition, there is a growing evidence that ginger leaves had higher antioxidant activity than rhizomes and flowers Eric Chan et al. [5]. Therefore, ginger leaves has great potential to be developed into functional foods and other health products.
